# Supplementary material for: Emissions and fate of organophosphate esters in outdoor urban environments
Source: Nat Commun. 2023 Mar 1;14:1175. doi: 10.1038/s41467-023-36455-7 (PMC9977944; doi:10.1038/s41467-023-36455-7)
Supplement: Supplementary file 1 — Supplementary Information [file 41467_2023_36455_MOESM1_ESM.pdf]

Supplementary Information For: Emissions and Fate of Organophosphate Esters in Outdoor Urban Environments

Timothy F. M. Rodgers[1], Amanda Giang[1*], Miriam L. Diamond[2,3], Emma Gillies[1], Amandeep Saini[4*]

[1]Institute for Resources, Environment and Sustainability, University of British Columbia, Vancouver Canada V6T 1Z4
[2]Department of Earth Sciences, University of Toronto, Toronto, Canada M5S 3B1
[3]School of the Environment, University of Toronto, Toronto, Canada M5S 3B1
[4]Air Quality Processes Research Section, Environment and Climate Change Canada, Toronto, Canada M3H5T4

Supplementary Results

S1. Model Evaluation

The surface water concentration predicted by the model, representing rivers in the modeled cities, was within the range of measured values reported in the literature, for 86% of predictions, and within a factor of 100 for 78% of predictions. Some of this model underestimation of water concentrations could be due to neglecting direct water emissions.[1] In the soil, 68% of predictions were within the range of measured values, and 79% within a factor of 100. The model also appeared to under-predict measured soil concentration, although this may be because many of the soil measurements were taken from areas where higher concentrations would be expected such as on roadsides[2,3] or near e-waste recycling facilities[4] compared with the model considering "average" conditions.

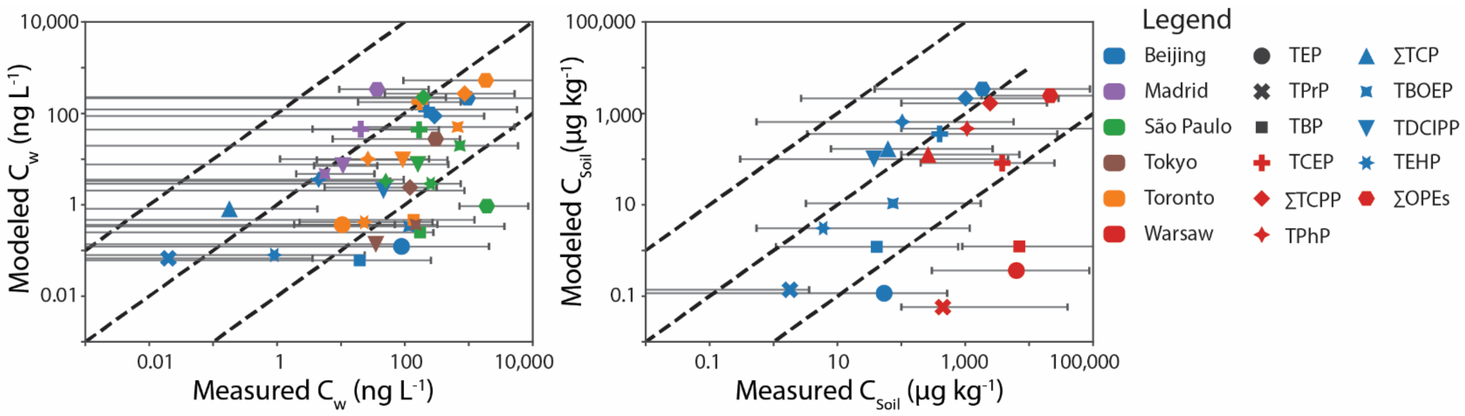

Supplementary Figure 1: Model evaluation. Literature measured mean vs model-predicted water (C_W, ng L^{-1}) and soil (C_{soil}, ug kg^{-1}) concentrations. Colors represent the city where the literature measurements were taken; shapes represent the concentration of each OPE. Error bars indicate the range of measured values; minimum values that were below the limit of detection are shown with the error bar extending to the left-hand side of the x-axis. Dashed lines indicate a 1:1 fit and modeled values 100 times larger or smaller than the literature values.

Table 1: Abbreviations, molecular weights, CAS numbers, and IUPAC names for the compounds investigated in this study

Abbreviation	Molecular Weight (g/mol)	CAS Number	IUPAC Name
TEP	182.16	78-40-0	triethyl phosphate
TPrP	224.23	513-08-6	tripropyl phosphate
TnBP	266.31	126-73-8	tributyl phosphate
TCEP	285.5	115-96-8	tris(2-chloroethyl)phosphate
TCiPP	327.6	13674-84-5	tris(chloroisopropyl)phosphate
TDCiPP	430.9	13674-87-8	tris(1,3-dichloroisopropyl)phosphate
TPhP	326.3	115-86-6	triphenyl phosphate
TmCP	368.4	563-04-2	tris(3-methylphenyl) phosphate
TBOEP	398.5	78-51-3	tris(2-butoxyethyl)phosphate
TEHP	434.6	78-42-2	tris(2-ethylhexyl)phosphate

Emissions fluxes for each city calculated with the different system boundaries were within a factor of 0.51-1.98 (Table 2). This is well within the expected ~order of magnitude uncertainty of the model,[1] indicating that fate processes were similar across a city's area, regardless of how the boundary was defined. The largest fluxes were estimated for the smallest (5km radius) boundary and the smallest for the larger 15 km buffer. The administrative areas, which tended to be larger than either, generally fell between the two buffer areas. Across all cities the average $\sum_{10}$OPEs varied by an average of <10% between boundaries, driven by the low sensitivity of TCEP and TCPP, which had the largest emissions.

Table 2: Summary statistics showing the sensitivity of modeled emissions fluxes to the choice of model boundary definition.

Boundaries	Min	Max	Mean	Median
5k/admin	0.64	1.98	1.16	1.10
15k/admin	0.51	1.38	0.95	0.99
5k/15k	0.80	1.60	1.23	1.25

The 2018 $\sum_{10}$OPEs estimate presented here for Toronto of 1,800 kg yr^{-1} was lower than the 3,300 kg yr^{-1} mean value presented in Rodgers et al.[1] for 2010 using the same model and boundary area. This near halving of emissions was caused by lower measured air concentrations; using the 2010 air concentrations with the 2018 parameterization gave an estimate of ~3,100 kg yr^{-1}. Emissions in 2010 and 2018 were dominated by TCEP which was designated as "harmful to human health" under the Canadian Chemicals Management Plan in 2013,[5] although the formal restrictions introduced on its use were limited to products intended for children under the age of three, which would likely not be sufficient to cause such a decrease.

He et al.[6] estimated OPE emissions across China using production data and activity-specific emissions factors. They estimated that in 2018 the province of Beijing emitted ~130,000 kg yr^{-1} or 7.3 µg m^{-2}, vs our estimate of ~5,100 kg yr^{-1} or 3.8 µg m^{-2} for the Beijing municipal area. Thus, we estimated ~2x lower emissions on an area-normalized basis based on similar measured air concentrations, despite the fact that our boundary contained a larger proportion of the urban area likely responsible for emissions. Thus, the difference in emissions intensity was likely caused by different estimations of chemical fate within the modeled domain.

S2. Emissions Regressions

We also found that emissions were mostly controlled by the magnitude of local sources rather than by transport limitations or environmental factors. Correlations (Supplementary Figure 2, Table S3) between the estimated emissions and the factors that our sensitivity analysis indicated were controls of contaminant fate (Supplementary Figure 3, SI Section S2) were generally not significant. The main exceptions were the relative humidity, ($p<0.05$ for TBOEP, TCIPP, TmCP, and $\sum_{10}$OPEs; r^2 from 0.17 – 0.30) and the precipitation rate ($p<0.05$ for TBOEP and TmCP; $r^2 = 0.20$ and 0.23, respectively) of the cities. Mechanically, increases in these variables wash contaminants out of the air, lowering the fugacity in the air compartment, which could lead to more emissions due to the larger fugacity gradient between the air and any products containing OPEs. We note that this effect would likely be small, and so this correlation may not imply causation.

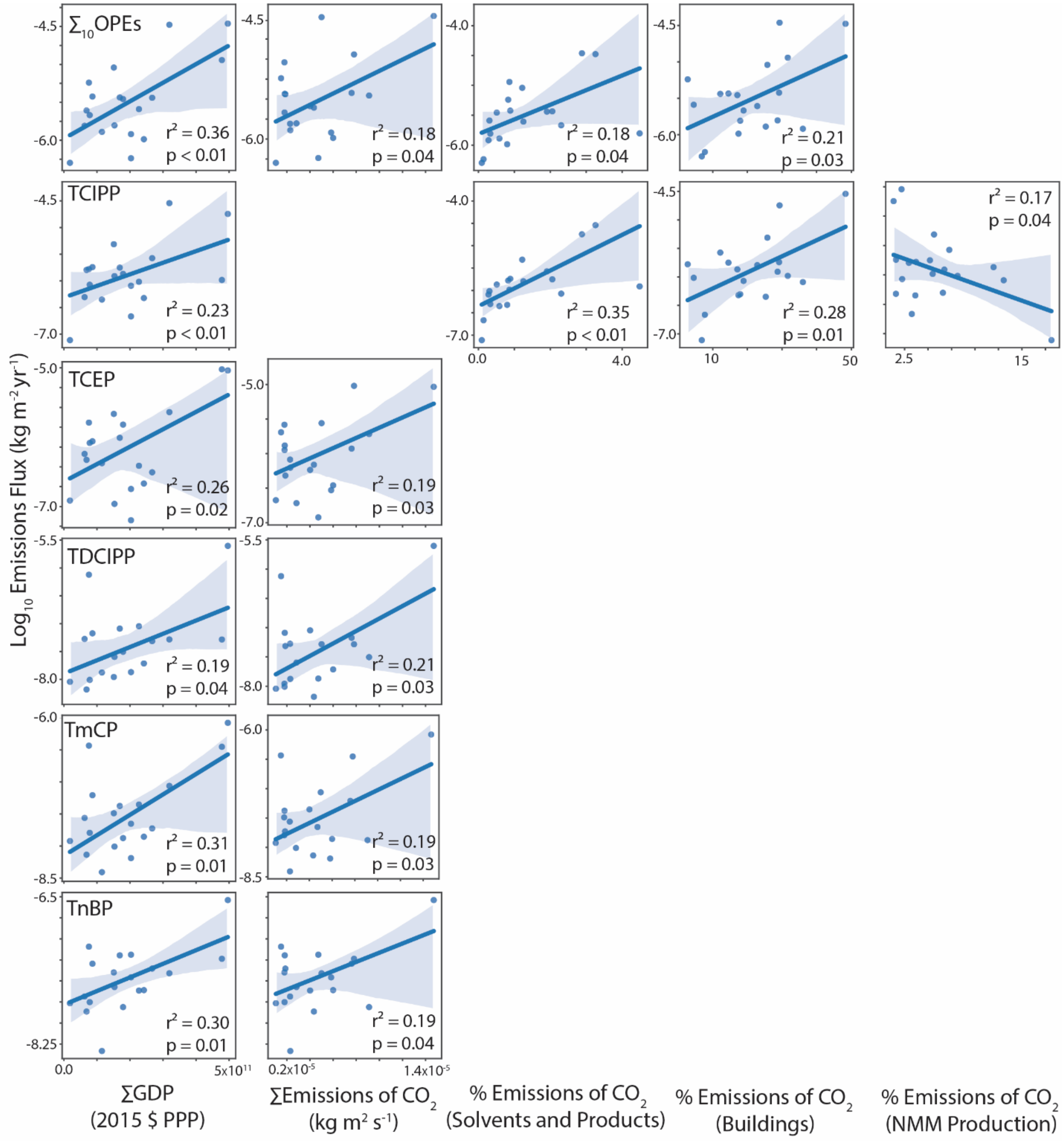

Supplementary Figure 2: Emissions Regressions. Regressions between emissions proxies (Gross Domestic Product (GDP) at 2015 purchasing power parity, anthropogenic emissions of greenhouse gases as CO_2 equivalents, and the proportion of anthropogenic CO_2 equivalent emissions from the Solvents and Products, Buildings and Non-Metallic Minerals (NMM) Production sectors, as described by EDGAR[7]) and the $\log_{10}$ transformed emissions flux (kg m^{-2} yr^{-1}). Selected regressions with $p<0.05$ are shown, r^2 is the adjusted correlation coefficient. Table S3 shows all regressions with $p<0.05$.

Table 3: Regressions with $p<0.05$ between emissions proxies (∑GDP, Gross Domestic Product (GDP) at 2015 purchasing power parity, GDP per capita at 2015 purchasing power parity, ∑CO_2 Emissions, anthropogenic emissions of greenhouse gases as CO_2 equivalents, and the proportion of anthropogenic CO_2 equivalent emissions from the Solvents and Products, Energy for Buildings, Solid Waste Incineration, Non-Metallic Minerals (NMM) Production, and Combustion for Manufacturing sectors, as described by EDGAR[7]), and transport controls (relative humidity %, precipitation rate mm/hr, area of vegetation m²) vs the log_{10} transformed emissions flux (kg m^{-2} yr^{-1}) for the specified OPE.

Regression	Independent Variable	m1	b	p_m1	p_b	adjr²	p_reg
**∑OPEs**	∑GDP	2.46E-12	-5.98	3.72E-03	1.94E-17	**0.36**	3.72E-03
**TCIPP**	%CO2 Solvents and Products	3.06E+01	-6.29	4.69E-03	7.00E-18	**0.35**	4.69E-03
**TBOEP**	RH	2.97E-02	-9.01	0.01	2.44E-09	**0.32**	0.01
**∑OPEs**	GDP per Capita	2.00E-05	-5.91	0.01	1.73E-17	**0.31**	0.01
**TmCP**	∑GDP	2.84E-12	-8.04	0.01	8.80E-18	**0.31**	0.01
**TnBP**	∑GDP	1.68E-12	-7.80	0.01	2.61E-21	**0.30**	0.01
**TCIPP**	%CO2 Energy for Buildings	2.89E+00	-6.50	0.01	2.23E-15	**0.28**	0.01
**TCEP**	GDP per Capita	1.90E-05	-6.42	0.02	1.32E-17	**0.26**	0.02
**TCEP**	∑GDP	2.19E-12	-6.45	0.02	3.31E-17	**0.26**	0.02
**∑OPEs**	RH	2.08E-02	-6.97	0.02	5.16E-10	**0.25**	0.02
**TEHP**	%CO2 Solid Waste Incineration	-1.41E+02	-8.40	0.03	3.57E-17	**0.24**	0.03
**TmCP**	GDP per Capita	2.22E-05	-7.95	0.02	9.05E-18	**0.24**	0.02
**TCIPP**	RH	2.35E-02	-7.54	0.02	1.97E-09	**0.23**	0.02
**TBOEP**	GDP per Capita	2.35E-05	-7.46	0.03	9.88E-15	**0.23**	0.03
**TCIPP**	∑GDP	2.37E-12	-6.34	0.02	4.57E-16	**0.23**	0.02
**TmCP**	Precipitation Rate	3.72E+00	-7.96	0.02	1.91E-17	**0.23**	0.02
**∑OPEs**	%CO2 Energy for Buildings	2.21E+00	-5.97	0.03	1.57E-15	**0.21**	0.03
**TDCIPP**	∑CO2 Emissions	9.46E+04	-7.81	0.03	2.35E-16	**0.21**	0.03
**TPrP**	Area of Vegetation	-1.10E-10	-7.41	0.03	3.42E-22	**0.21**	0.03
**TBOEP**	Precipitation Rate	4.04E+00	-7.44	0.04	1.69E-14	**0.20**	0.04
**TCEP**	∑CO2 Emissions	7.49E+04	-6.38	0.03	2.94E-17	**0.19**	0.03
**TDCIPP**	∑GDP	2.40E-12	-7.84	0.04	6.71E-16	**0.19**	0.04
**TnBP**	∑CO2 Emissions	5.29E+04	-7.72	0.04	4.30E-21	**0.19**	0.04
**TCIPP**	GDP per Capita	1.88E-05	-6.27	0.04	3.48E-16	**0.18**	0.04
**∑OPEs**	%CO2 Solvents and Products	2.02E+01	-5.78	0.04	1.53E-17	**0.18**	0.04
**∑OPEs**	∑CO2 Emissions	7.01E+04	-5.83	0.04	9.76E-17	**0.18**	0.04
**TCIPP**	%CO2 NMM Production	-6.28E+00	-5.53	0.04	2.74E-15	**0.17**	0.04
**TmCP**	RH	2.21E-02	-9.05	0.04	5.21E-10	**0.17**	0.04
**TmCP**	∑CO2 Emissions	8.56E+04	-7.89	0.04	2.14E-17	**0.17**	0.04
**TEP**	%CO2 Combustion for Manufacturing	-2.22E+00	-7.23	0.05	1.98E-16	**0.17**	0.05
**TPhP**	∑GDP	1.95E-12	-7.38	0.05	2.44E-17	**0.16**	0.05

S3. Elemental Effects Sensitivity

We conducted a global "Elemental Effects" sensitivity analysis[8] to elucidate the processes governing modeled emissions, represented by the emissions flux (kg m^{-2} yr^{-1}), and chemical fate represented by the proportion of emissions advected from the system (%) in outdoor urban environments (Supplementary

Figure 3). As expected, modeled emissions flux was most sensitive to the target air concentration, while compound fate was not (Supplementary Figure 3, left side). Both outputs were least sensitive to “generic” parameters, such as mass-transport coefficients that remained the same across cities and chemicals (Supplementary Figure 3, right side).

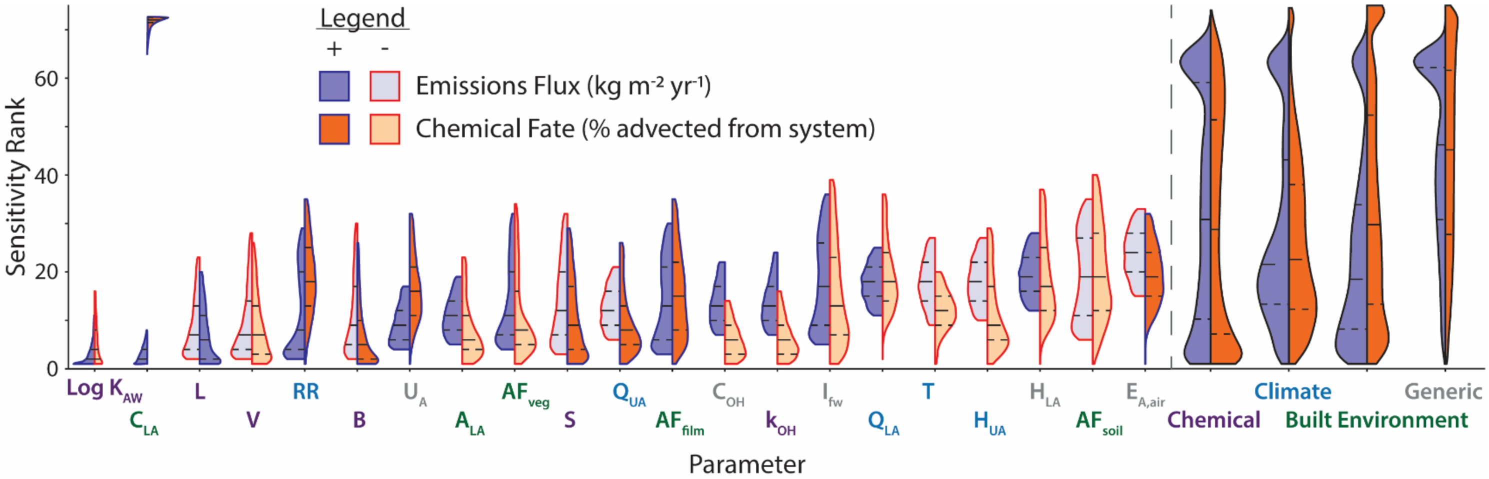

Supplementary Figure 3: Violinplots showing the rank-transformed sensitivity of the modeled emissions flux (purple) and chemical fate (orange) to the input parameters. The font color represents the parameter groupings, with purple font representing chemical-specific parameters; blue font, climate; green font, built-environment; and grey font, generic parameters. Red outlines with a lighter shade and blue outlines with a darker shade indicate that a higher parameter value lowered or raised the output magnitude, respectively. Horizontal lines in each violinplot represent quartiles, and label colors represent the parameter category (as shown on the right-hand side). The middle 80% distribution for each parameter is shown, from a run of 10,000 random trials. The overall distribution for all parameters in each category is shown on the right. Abbreviations are as follows: concentration in the lower air (C_{LA}), air-water partition coefficient (log K_{AW}), Abraham’s solvation parameters (L, V, B, S), upper-lower air mixing rate ($U_{A)}$, area of lower air (A_{LA}), rain rate (RR), flow in compartment j (Q_j), concentration I and reaction rate (k) of hydroxyl, ratio of area in compartment j to lower air (AF_j), height of compartment j (H_J), temperature (T), wet interception fraction (I_{fw}), and activation energy in the air ($E_{A,air}$)

Overall, the sensitivity analysis showed that chemical fate in the urban environment varied substantially across cities depending on interactions between the built environment, a chemical’s physicochemical properties, and the city’s climate. The strong influence of the target air concentration supports the contention that emissions of OPEs are driven by the intensity of local emissions and not “transport” factors, such as increases in vapor pressure at higher temperatures that would cause greater emissions.[9,10] However, the measured air concentration is itself known to vary substantial in the course of a year;[9,11] our results suggest that this is due to the sensitivity of the modeled emissions to chemical fate, with higher emissions necessary to achieve an observed air concentration in some urban environments vs others.

For the built environment, chemicals fate was most sensitive to the area of the city’s footprint, and to the area-factors (A_j/A_{city}, $m^2\ m^{-2}$) of vegetation and film within that footprint. For physicochemical properties, the most important parameter was the air-water partition coefficient (log K_{AW}), followed by three solute descriptors used in calculating partition coefficients from ppLFERs (L, V and B). The hexadecane-air partition coefficient (L) and the molar volume (V) represent cavitation and other non-polar forces in partitioning systems, while the hydrogen bonding basicity (B) represents polar forces for OPEs (where the hydrogen-bonding acidity is generally negligible). This indicates that, as expected,

differences between specific chemical fates in the same urban environment are influenced by the “three solubilities” of water, organics and air; and could be well-described by comparing the fate of PMT vs PBT compounds.

Finally, for climate, the most important parameters were the rain rate (RR); the rates of advection in the upper and lower air compartments (Q_{LA} and Q_{UA}), respectively, which are determined by the wind speed; the air temperature; and the height of the upper air compartment, which is modeled as the planetary boundary layer height.

Certain parameters had non-normal rank sensitivity distributions, indicating parameter interactions. For instance, the 5th most sensitive factor for emissions was the rain rate (RR), with the parameter’s rank sensitivity showing a bimodal distribution indicating that emissions of more soluble species or those that were entirely particle-bound were very sensitive to the rain rate, while more volatile, less soluble chemicals were less so. This highlights the need for a global sensitivity analysis for even relatively simple multimedia models.

The interaction between which parameters increased or decreased either emissions, total (air and water) advection, or both simultaneously, highlights the complex, interdependent nature of chemical fate in the urban environment. Although an increased flow rate in the upper and lower air directly increased both emissions and advection, for many other parameters, higher values increased emissions while decreasing advection, or vice-versa. For instance, the area factor of vegetation (AF_{veg}) had a large positive impact on emissions and a negative impact on total advection, indicating that larger emissions are driven by atmospheric deposition and subsequent transformation in the plant compartment. By contrast, increasing the area of the urban film (AF_{film}) or soil (AF_{soil}) increased both emissions and advection. In this case, increasing the area of the film or soil compartment increased atmospheric deposition and subsequent washoff to and advection from the water compartment. These parameters have bimodal sensitivity rank distributions for the emissions flux, similarly to the rain rate, because volatile lower-solubility compounds were less susceptible to washoff.

Supplementary Methods

Impervious Surface Index

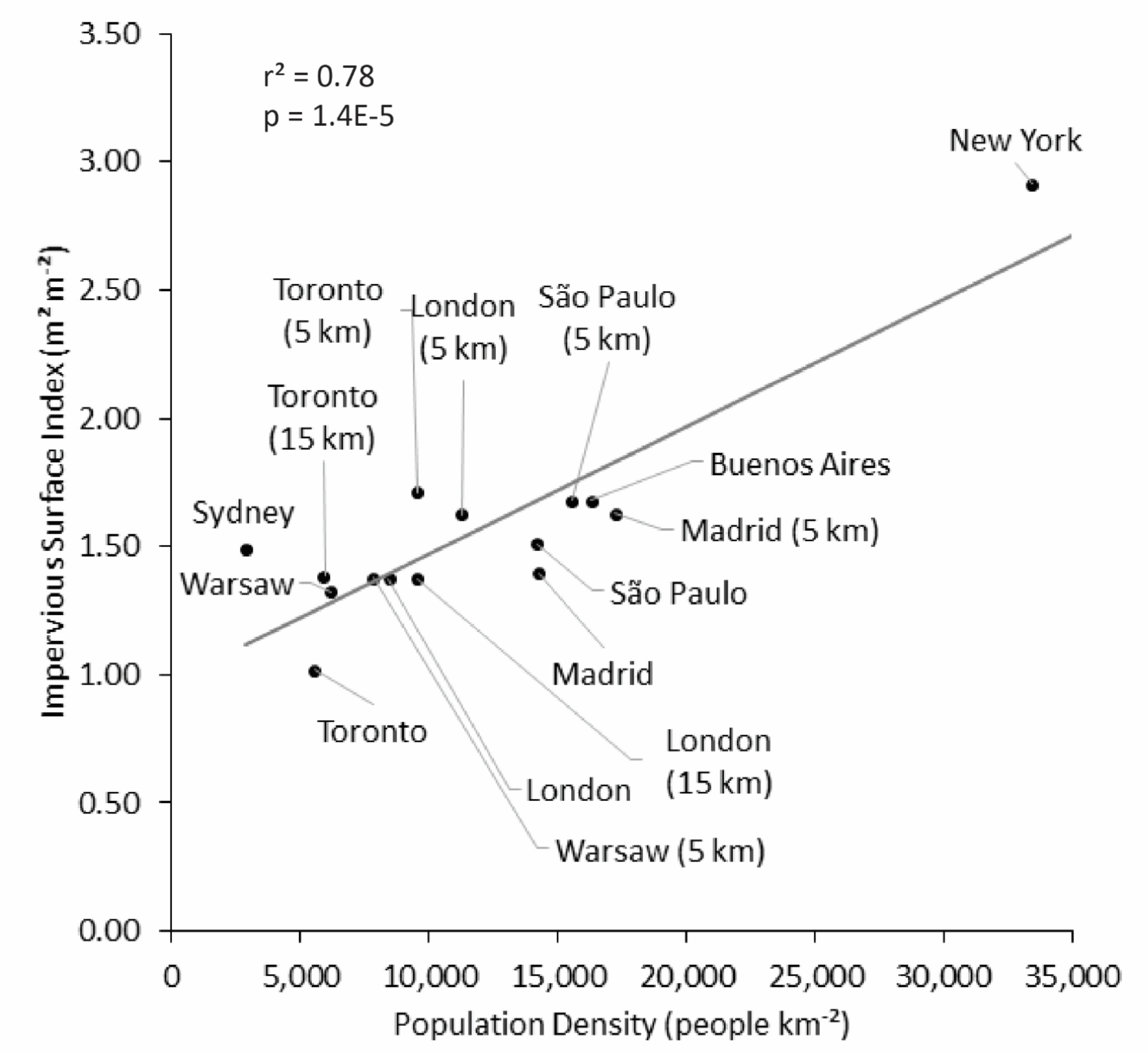

Supplementary Figure 4: Impervious surface index regression plot. Relationship between the impervious surfaces index and population density in the built-up area (as described in the Methods). The brackets indicate the model boundary parameterization represented in each point, a lack of brackets indicates the city administrative area.

Additional Supplementary Figures

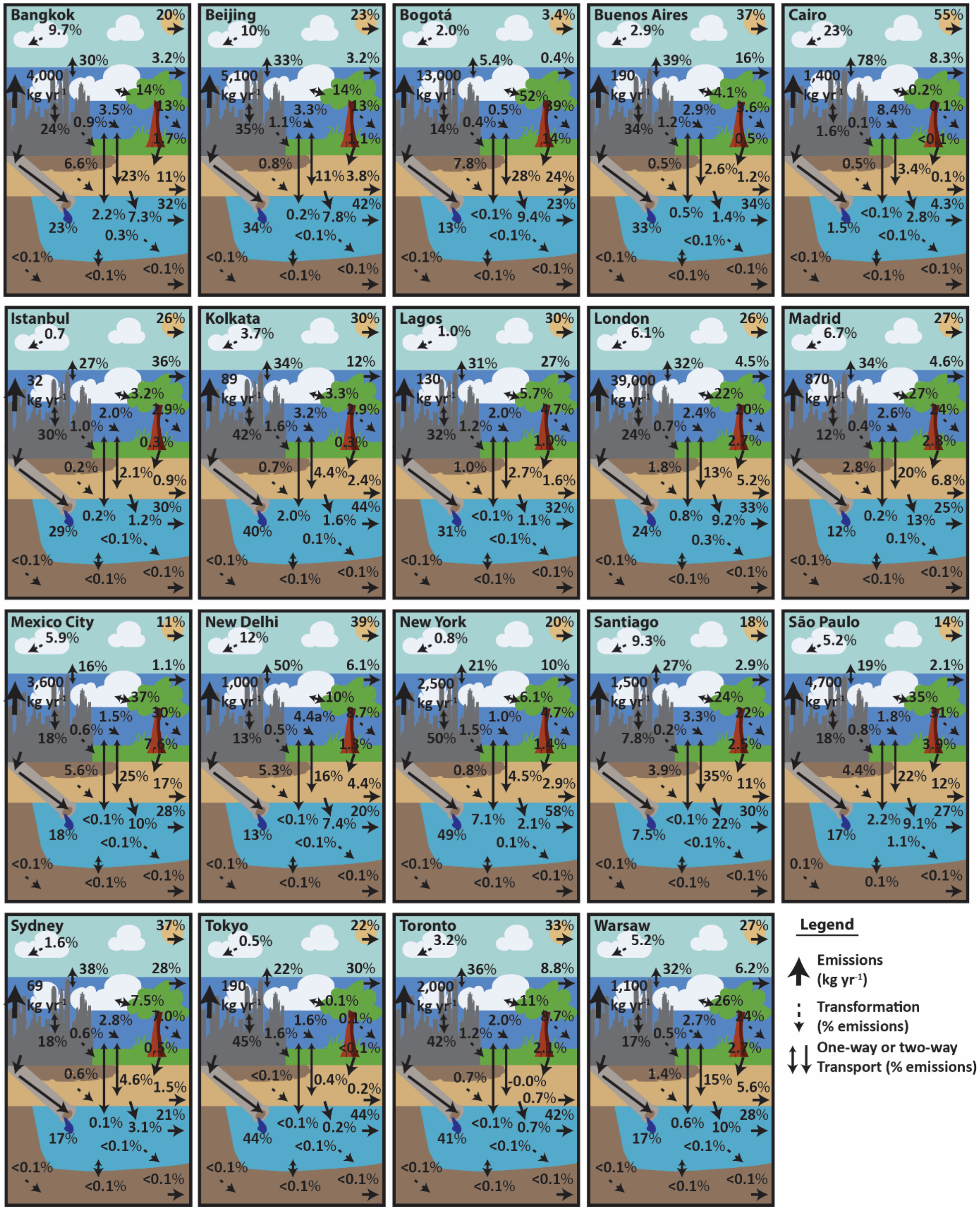

Supplementary Figure 5: Schematic diagrams showing the $\sum_{10}$OPEs emissions and fate for each of the modeled cities using the 2018 annual average administrative area baseline model fate parameterization. Advective processes leaving the city's modeled boundary are shown with one-way transport arrows at the right-hand side of the Supplementary Figure for the upper air, lower air, soil (to groundwater), water, and sediment (burial) compartments. Supplementary Figures may not sum exactly to 100 due to rounding. Supplementary Figures S5-S15 show the fates of each individual compound across the 19 cities. The trees, grass tufts, clouds, and city skylines in the model fate diagram were generated with the assistance of DALL·E 2.[12]

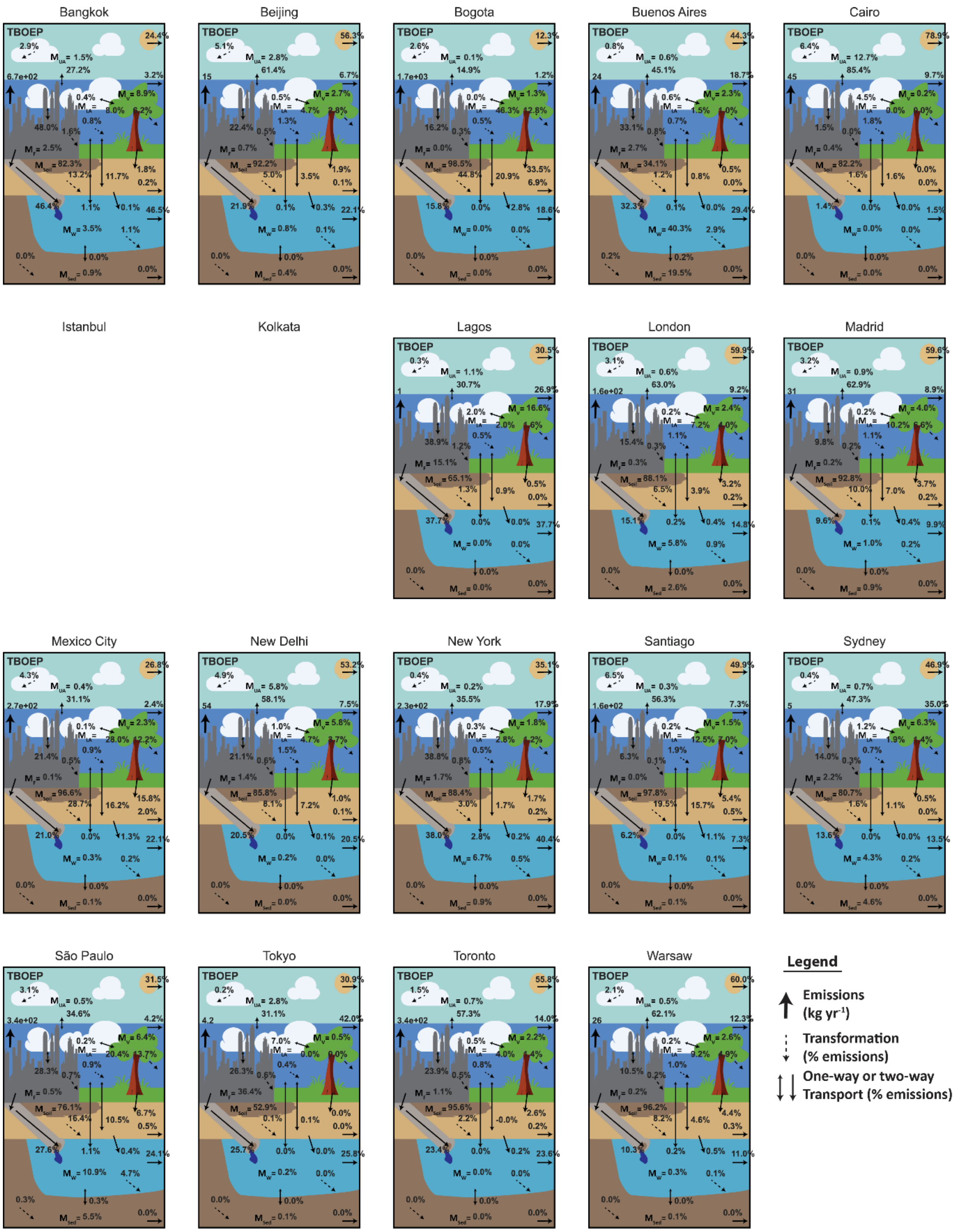

Supplementary Figure 6: TBOEP emissions and fate for each of the modeled cities using the 2018 annual average administrative area baseline model fate parameterization. Advective processes leaving the city's modeled boundary are shown with one-way transport arrows at the right-hand side of the Supplementary Figure for the upper air, lower air, soil (to groundwater), water, and sediment (burial) compartments. Blanks show cities where no TBOEP was emitted. Supplementary Figures may not sum exactly to 100 due to rounding. The trees, grass tufts, clouds, and city skylines in the model fate diagram were generated with the assistance of DALL·E 2.[12]

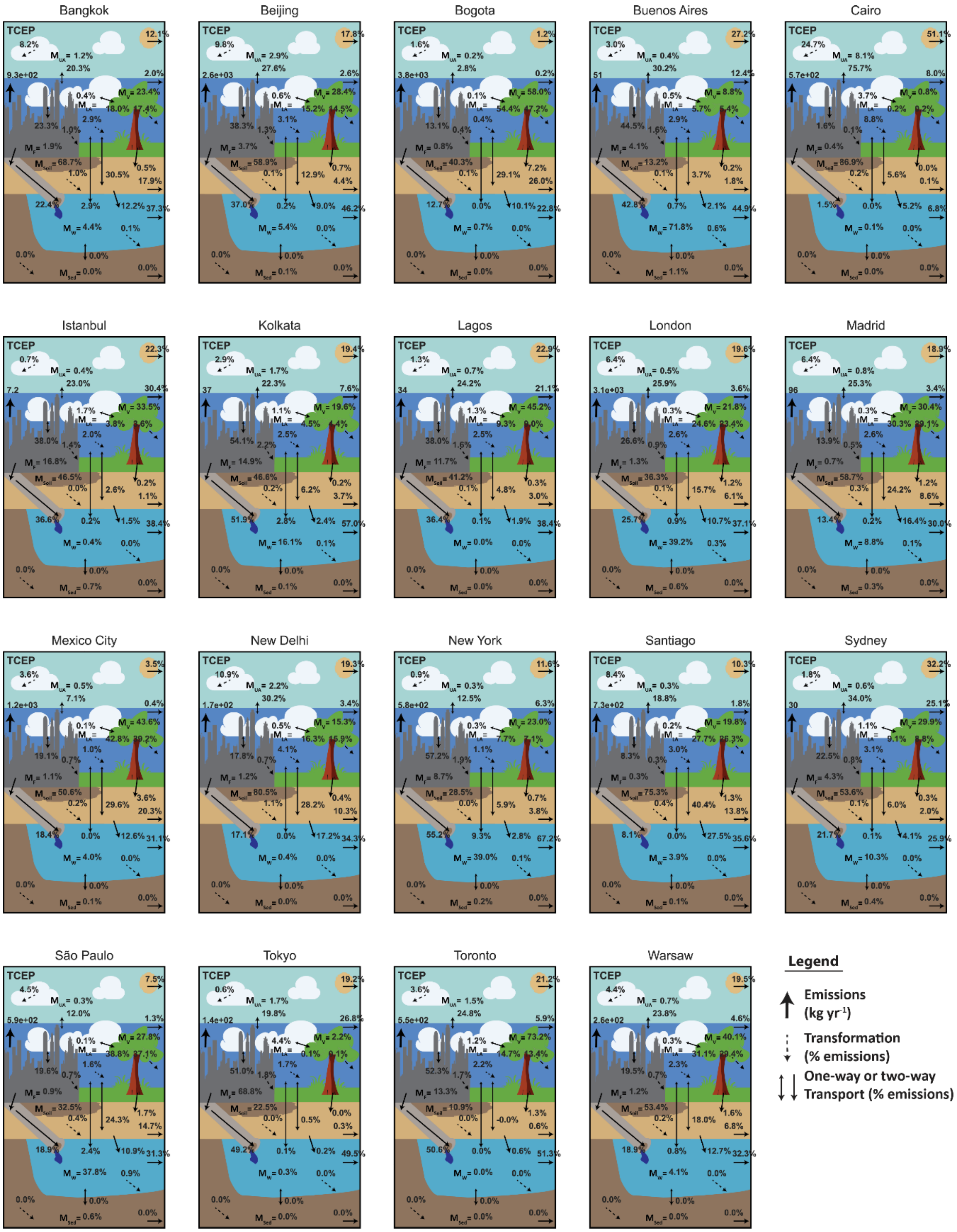

Supplementary Figure 7: TCEP emissions and fate for each of the modeled cities using the 2018 annual average administrative area baseline model fate parameterization. Advective processes leaving the city's modeled boundary are shown with one-way transport arrows at the right-hand side of the Supplementary Figure for the upper air, lower air, soil (to groundwater), water, and sediment (burial) compartments. Supplementary Figures may not sum exactly to 100 due to rounding. The trees, grass tufts, clouds, and city skylines in the model fate diagram were generated with the assistance of DALL·E 2.[12]

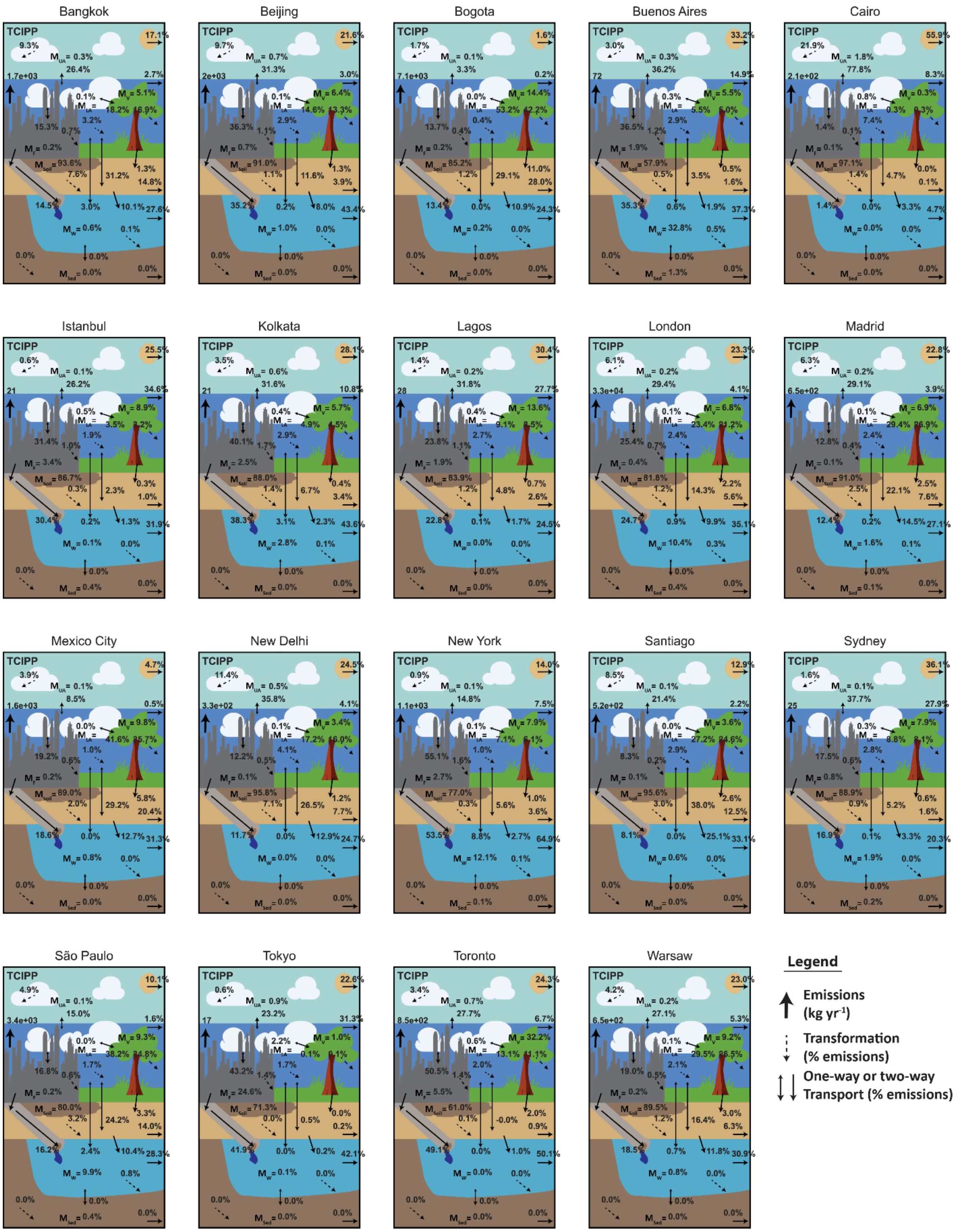

Supplementary Figure 8: TCIPP emissions and fate for each of the modeled cities using the 2018 annual average administrative area baseline model fate parameterization. Advective processes leaving the city's modeled boundary are shown with one-way transport arrows at the right-hand side of the Supplementary Figure for the upper air, lower air, soil (to groundwater), water, and sediment (burial) compartments. Supplementary Figures may not sum exactly to 100 due to rounding. The trees, grass tufts, clouds, and city skylines in the model fate diagram were generated with the assistance of DALL·E 2.[12]

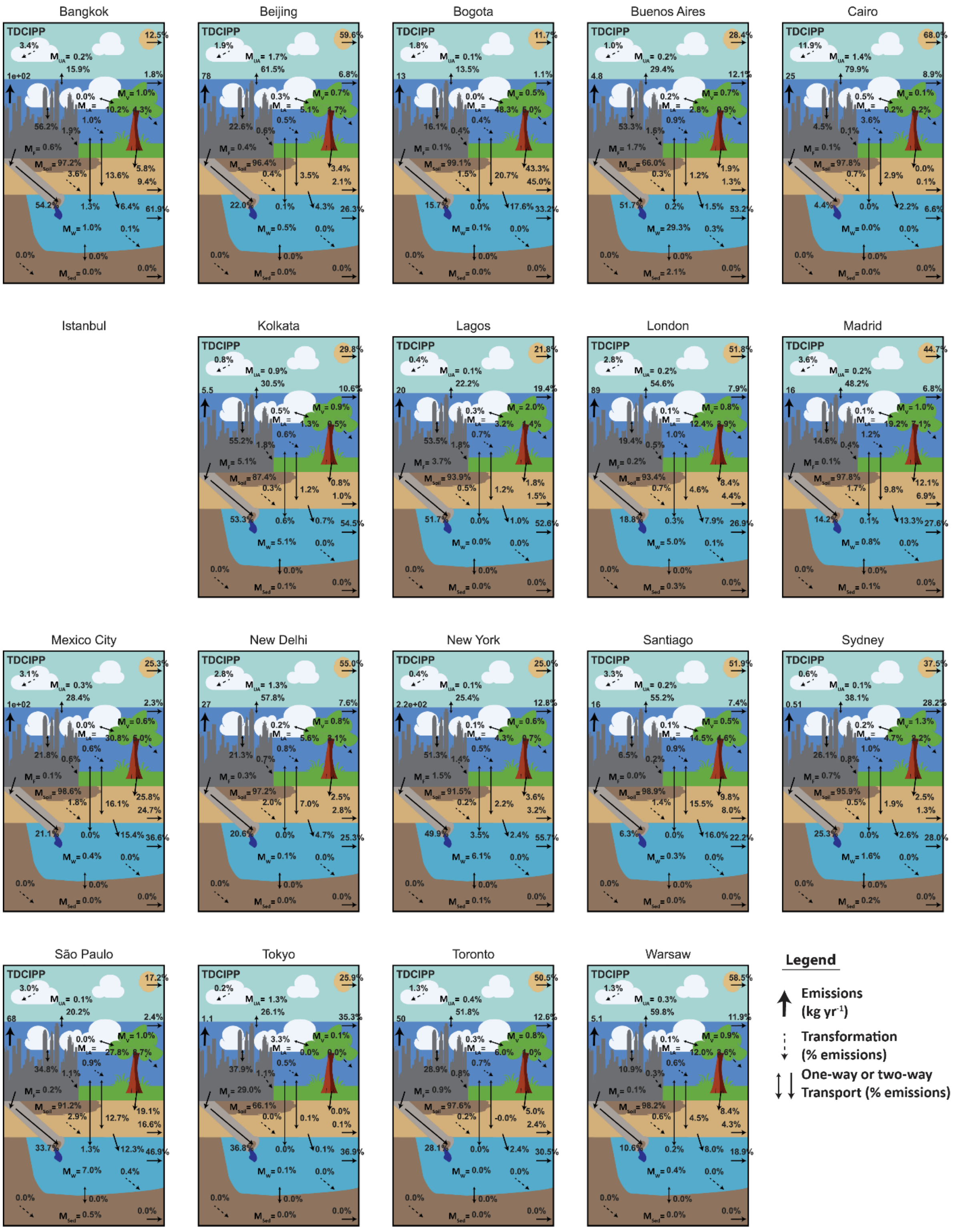

Supplementary Figure 9: TDCIPP emissions and fate for each of the modeled cities using the 2018 annual average administrative area baseline model fate parameterization. Advective processes leaving the city's modeled boundary are shown with one-way transport arrows at the right-hand side of the Supplementary Figure for the upper air, lower air, soil (to groundwater), water, and sediment (burial) compartments. Supplementary Figures may not sum exactly to 100 due to rounding. The trees, grass tufts, clouds, and city skylines in the model fate diagram were generated with the assistance of DALL·E 2.[12] Blanks show cities where no TDCIPP was emitted.

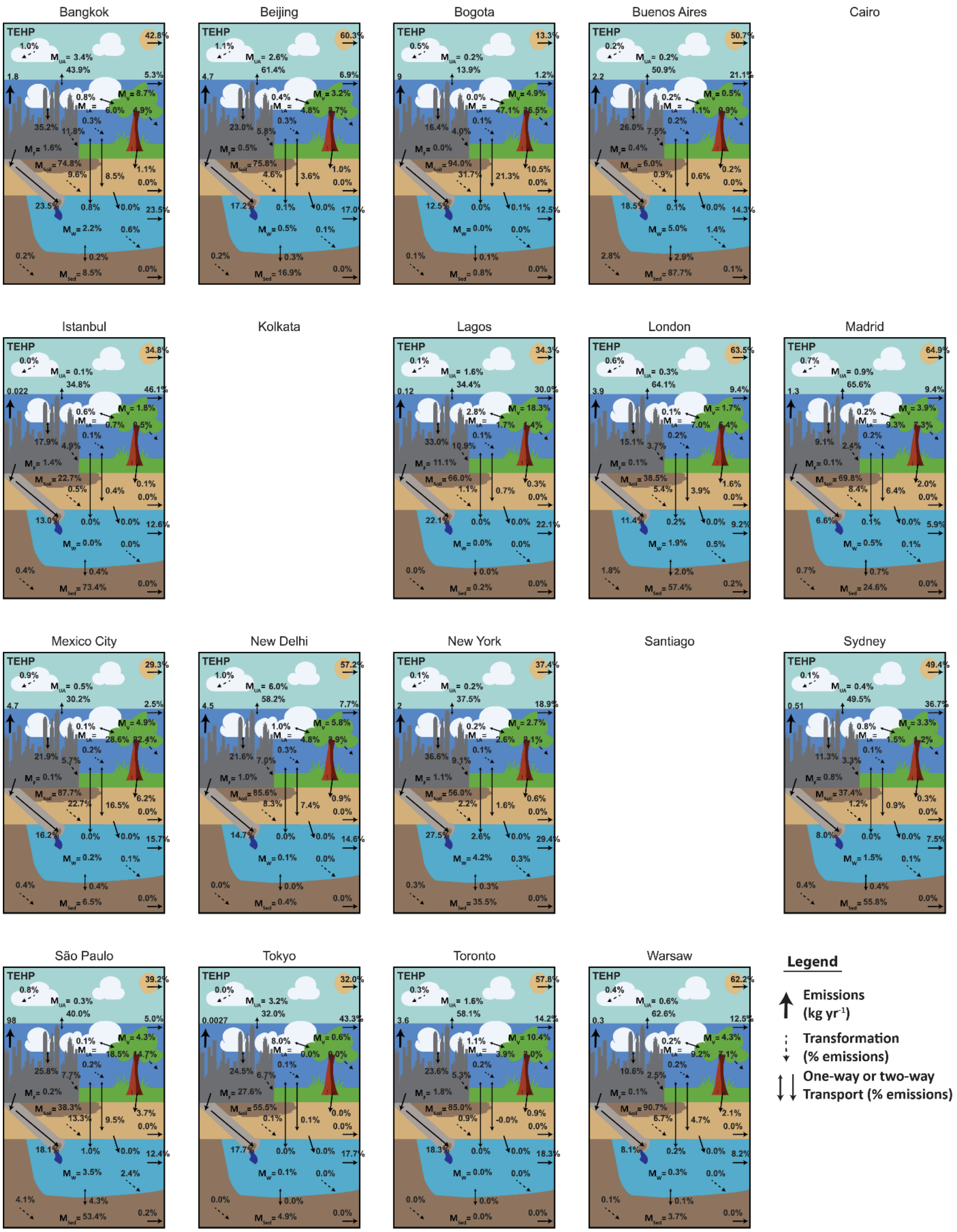

Supplementary Figure 10: TEHP emissions and fate for each of the modeled cities using the 2018 annual average administrative area baseline model fate parameterization. Advective processes leaving the city's modeled boundary are shown with one-way transport arrows at the right-hand side of the Supplementary Figure for the upper air, lower air, soil (to groundwater), water, and sediment (burial) compartments. Supplementary Figures may not sum exactly to 100 due to rounding. The trees, grass tufts, clouds, and city skylines in the model fate diagram were generated with the assistance of DALL·E 2.[12] Blanks show cities where no TEHP was emitted.

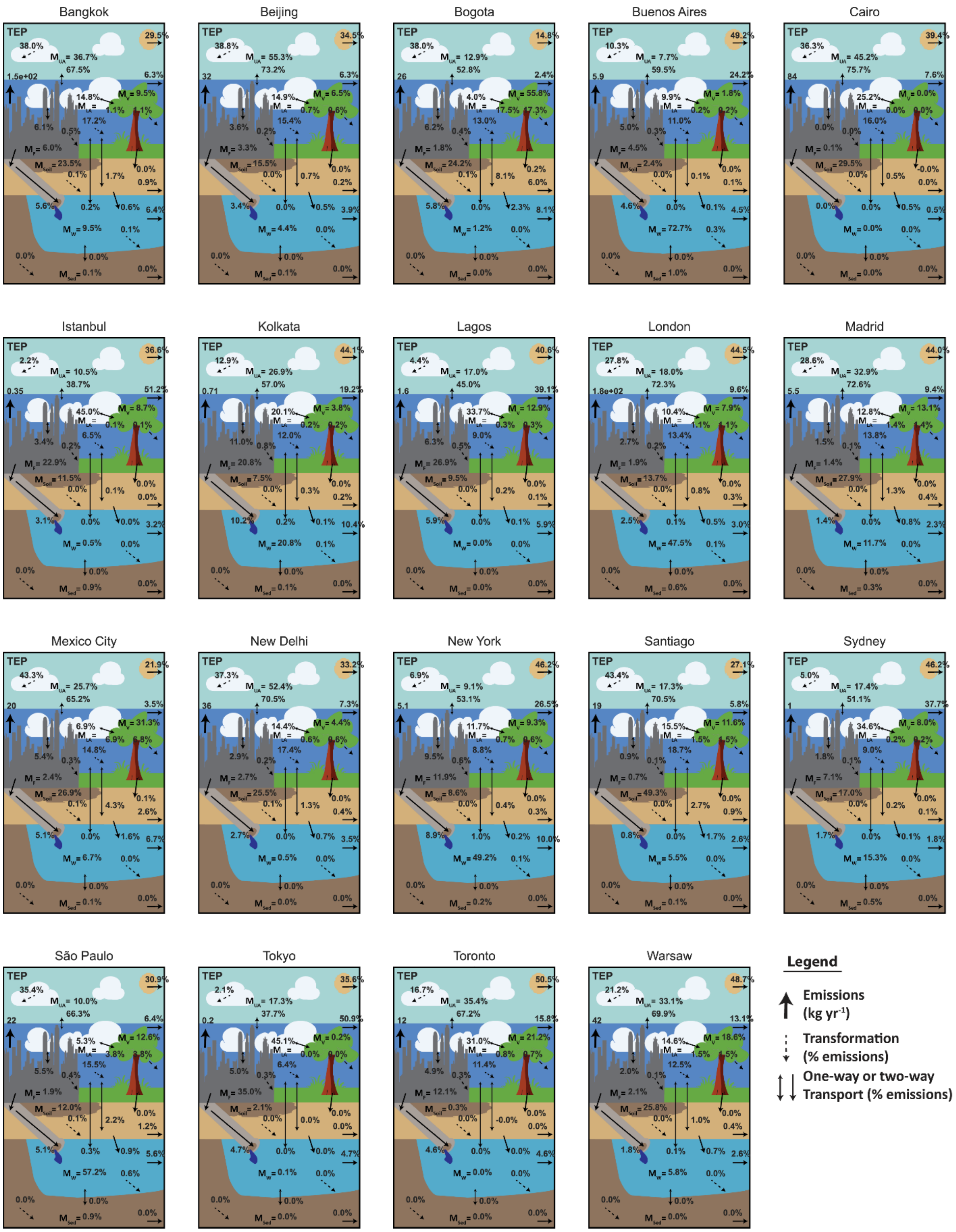

Supplementary Figure 11: TEP emissions and fate for each of the modeled cities using the 2018 annual average administrative area baseline model fate parameterization. Advective processes leaving the city's modeled boundary are shown with one-way transport arrows at the right-hand side of the Supplementary Figure for the upper air, lower air, soil (to groundwater), water, and sediment (burial) compartments. Supplementary Figures may not sum exactly to 100 due to rounding. The trees, grass tufts, clouds, and city skylines in the model fate diagram were generated with the assistance of DALL·E 2.[12]

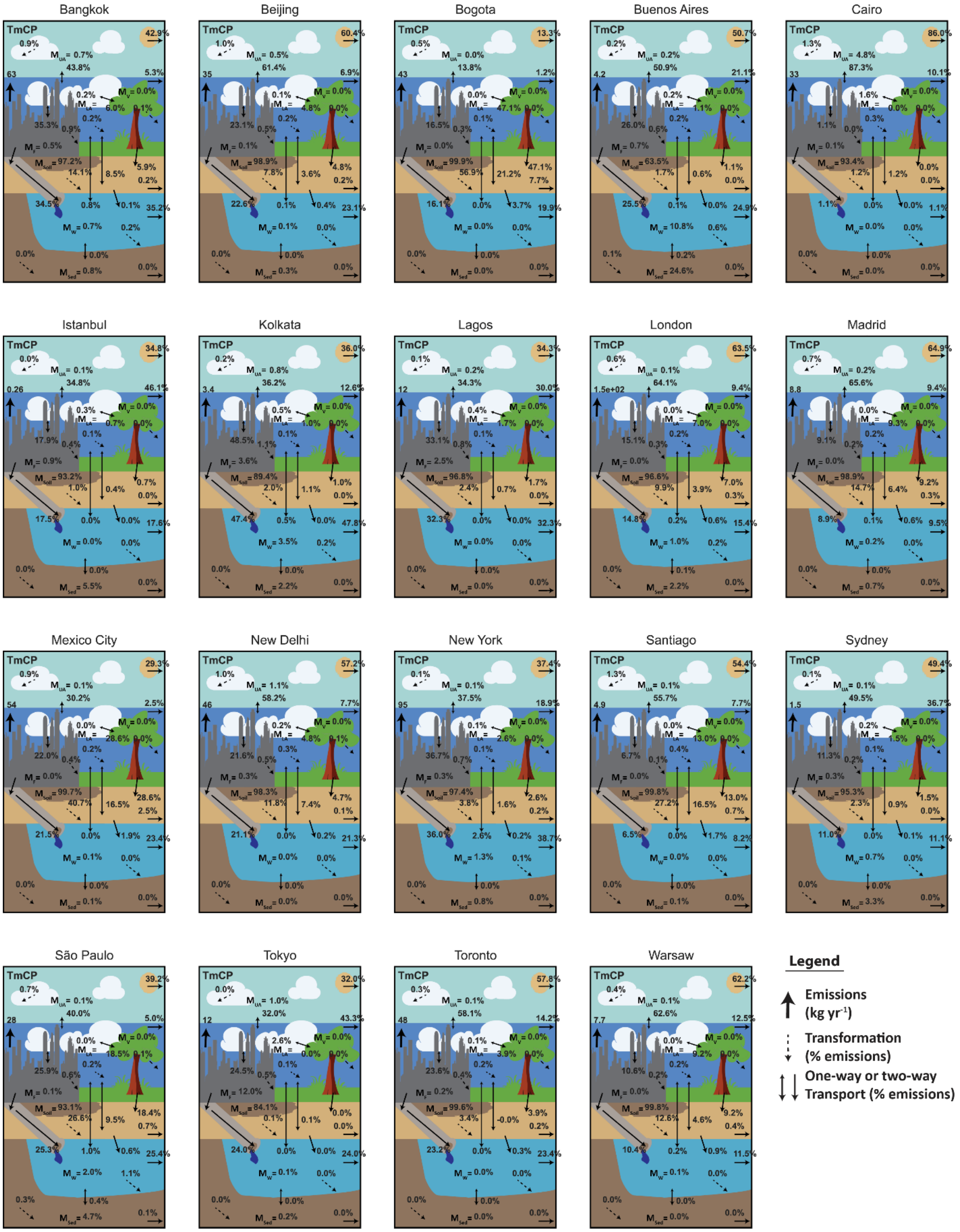

Supplementary Figure 12: TmCP emissions and fate for each of the modeled cities using the 2018 annual average administrative area baseline model fate parameterization. Advective processes leaving the city's modeled boundary are shown with one-way transport arrows at the right-hand side of the Supplementary Figure for the upper air, lower air, soil (to groundwater), water, and sediment (burial) compartments. Supplementary Figures may not sum exactly to 100 due to rounding. The trees, grass tufts, clouds, and city skylines in the model fate diagram were generated with the assistance of DALL·E 2.[12]

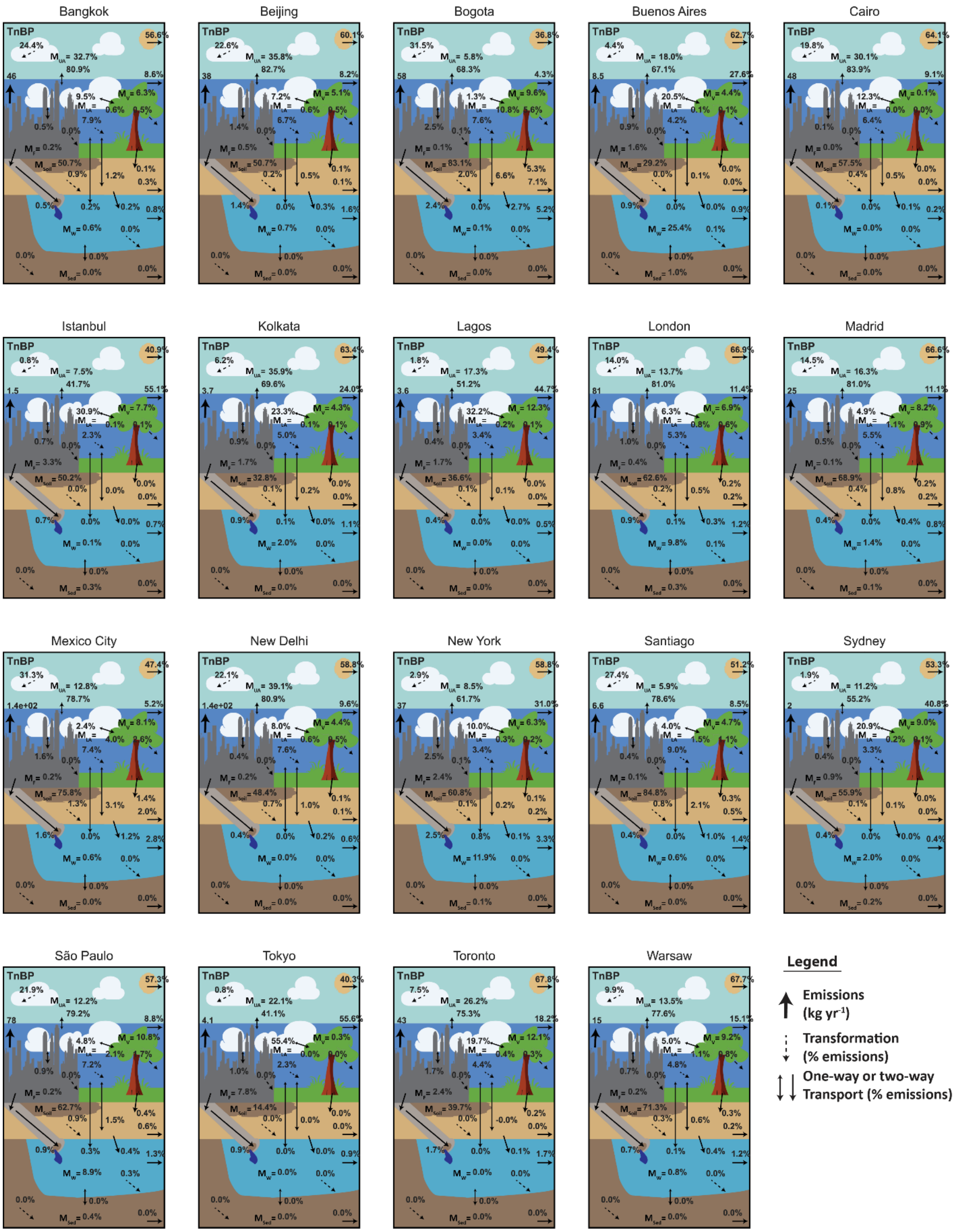

Supplementary Figure 13: TnBP emissions and fate for each of the modeled cities using the 2018 annual average administrative area baseline model fate parameterization. Advective processes leaving the city's modeled boundary are shown with one-way transport arrows at the right-hand side of the Supplementary Figure for the upper air, lower air, soil (to groundwater), water, and sediment (burial) compartments. Supplementary Figures may not sum exactly to 100 due to rounding. The trees, grass tufts, clouds, and city skylines in the model fate diagram were generated with the assistance of DALL·E 2.[12]

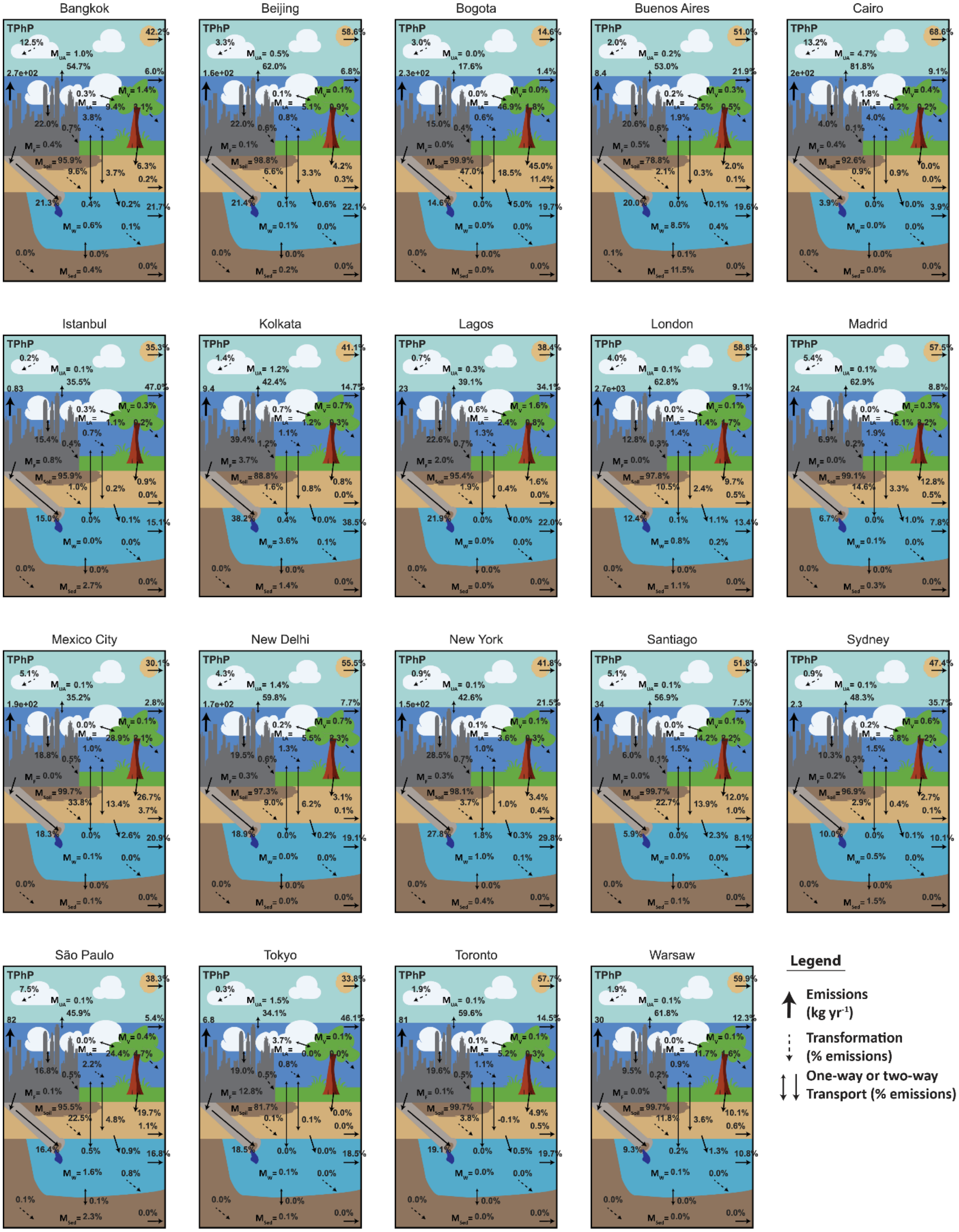

Supplementary Figure 14: TPhP emissions and fate for each of the modeled cities using the 2018 annual average administrative area baseline model fate parameterization. Advective processes leaving the city's modeled boundary are shown with one-way transport arrows at the right-hand side of the Supplementary Figure for the upper air, lower air, soil (to groundwater), water, and sediment (burial) compartments. Supplementary Figures may not sum exactly to 100 due to rounding. The trees, grass tufts, clouds, and city skylines in the model fate diagram were generated with the assistance of DALL·E 2.[12]

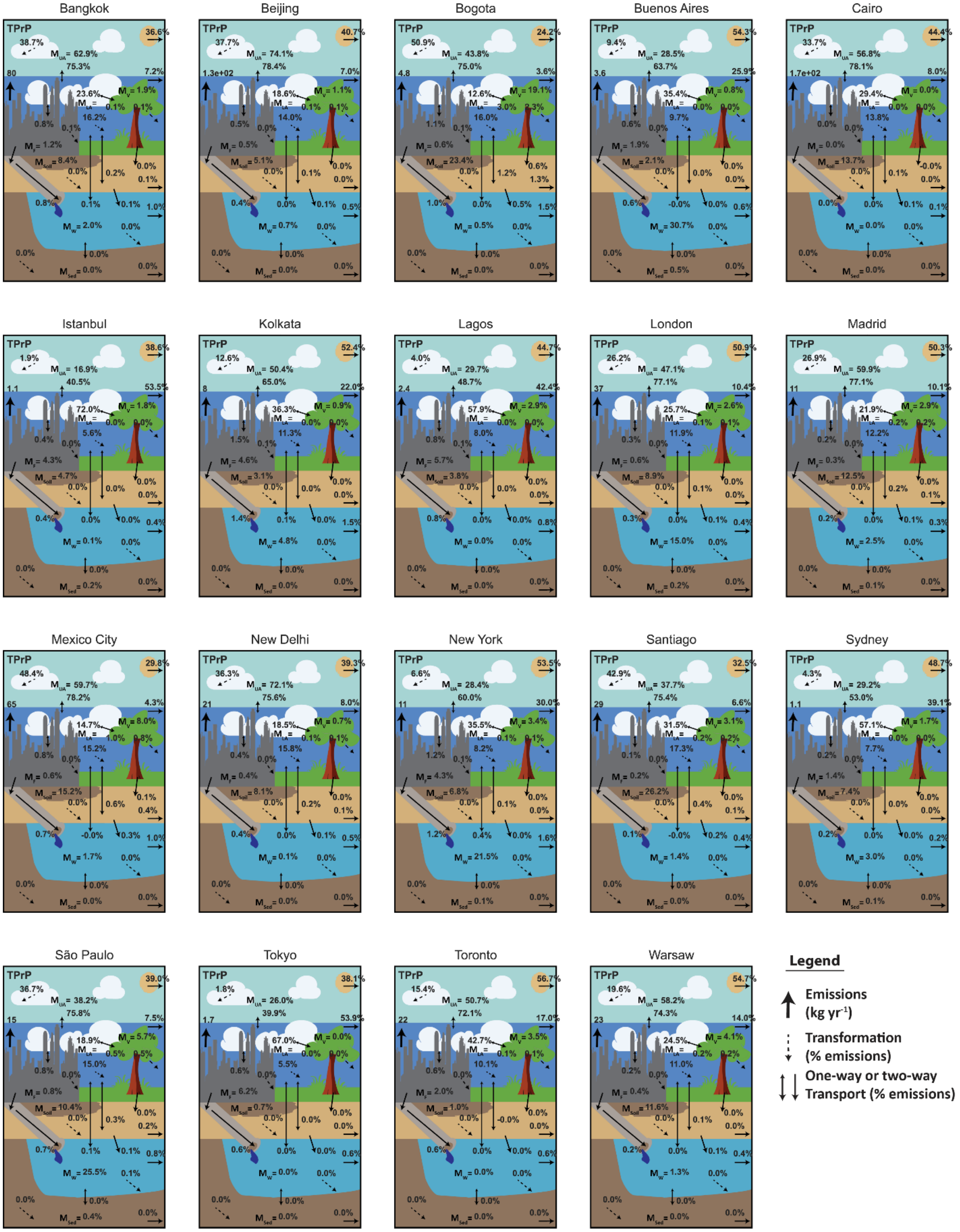

Supplementary Figure 15: TPrP emissions and fate for each of the modeled cities using the 2018 annual average administrative area baseline model fate parameterization. Advective processes leaving the city's modeled boundary are shown with one-way transport arrows at the right-hand side of the Supplementary Figure for the upper air, lower air, soil (to groundwater), water, and sediment (burial) compartments. Supplementary Figures may not sum exactly to 100 due to rounding. The trees, grass tufts, clouds, and city skylines in the model fate diagram were generated with the assistance of DALL·E 2.[12] Blanks show cities where no TEHP was emitted.

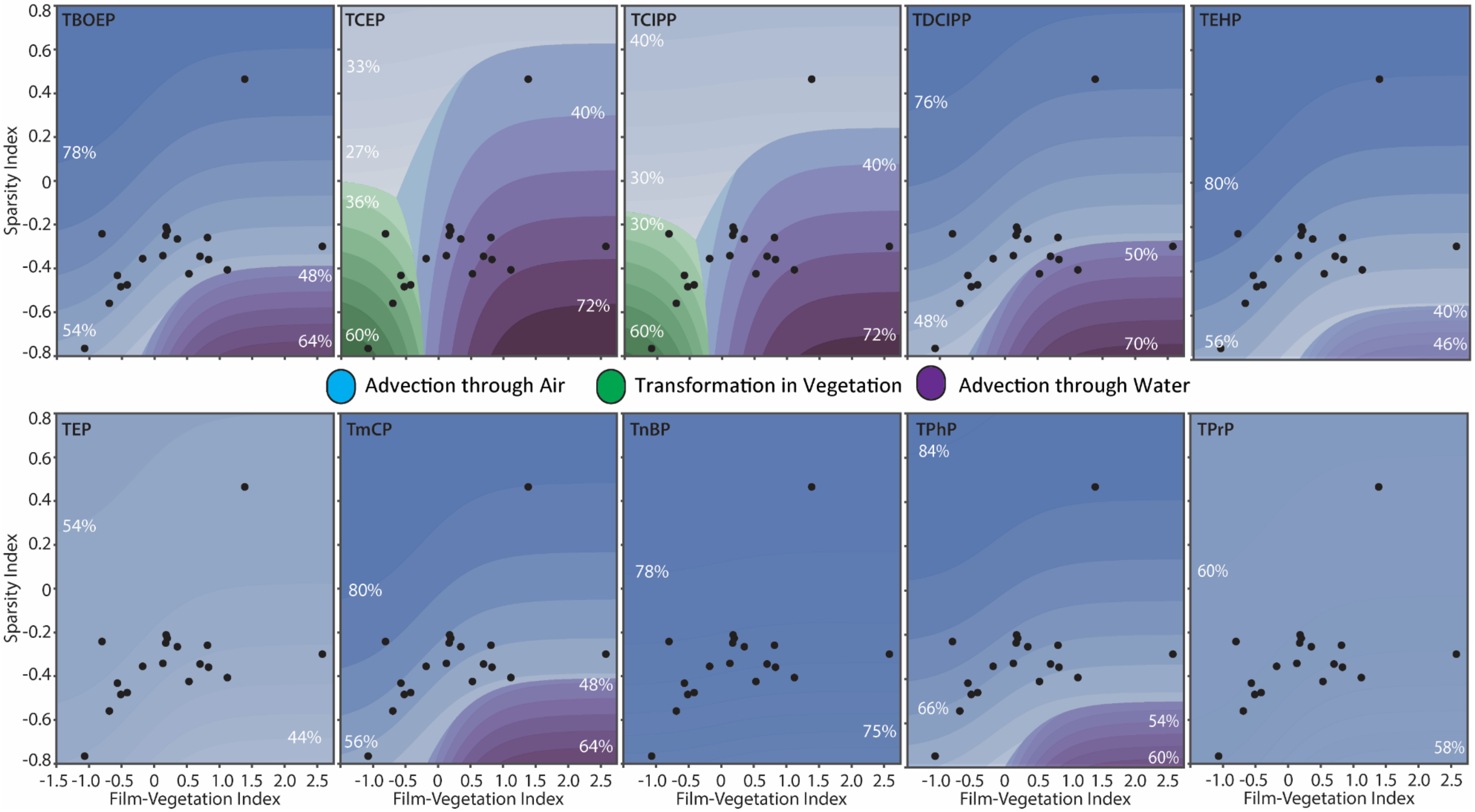

Supplementary Figure 16: City-space diagrams showing the fate of each OPE across the built-environment "sparsity" and "film-vegetation" indices. Models were parameterized using the "average" city, as explained in the main text. The color of the contour lines shows the dominant fate processes, with the intensity of the color showing the percentage of total emissions represented by the dominant fate (as shown by the percentage values in white text

Supplementary References

1. Rodgers, T. F. M., Truong, J. W., Jantunen, L. M., Helm, P. A. & Diamond, M. L. Organophosphate Ester Transport, Fate, and Emissions in Toronto, Canada, Estimated Using an Updated Multimedia Urban Model. *Environ. Sci. Technol.* **52,** 12465–12474 (2018).
2. Fabiańska, M. J., Kozielska, B., Konieczyński, J. & Bielaczyc, P. Occurrence of organic phosphates in particulate matter of the vehicle exhausts and outdoor environment – A case study. *Environ. Pollut.* **244,** 351–360 (2019).
3. Li, W., Shi, Y., Gao, L., Wu, C., Liu, J. & Cai, Y. Occurrence, distribution and risk of organophosphate esters in urban road dust in Beijing, China. *Environ. Pollut.* **241,** 566–575 (2018).
4. Wang, Y., Sun, H., Zhu, H., Yao, Y., Chen, H., Ren, C., Wu, F. & Kannan, K. Occurrence and distribution of organophosphate flame retardants (OPFRs) in soil and outdoor settled dust from a multi-waste recycling area in China. *Sci. Total Environ.* **625,** 1056–1064 (2018).
5. CEPA. Order 2013-87-01-01 Amending the Domestic Substances List persuant to subsection 87(3) of the act. (2013).
6. He, J., Wang, Z., Zhao, L., Ma, H., Huang, J., Li, H., Mao, X., Huang, T., Gao, H. & Ma, J. Gridded emission inventory of organophosphorus flame retardants in China and inventory validation. *Environmental Pollution* **290,** 118071 (2021).
7. European Commission. Joint Research Centre. Institute for Environment and Sustainability. *Global emission inventories in the Emission Database for Global Atmospheric Research (EDGAR): manual (I) gridding: EDGAR emissions distribution on global gridmaps.* (Publications Office, 2012). at <https://data.europa.eu/doi/10.2788/81454>
8. Saltelli, A., Ratto, M., Andres, T., Campolongo, F., Cariboni, J., Gatelli, D., Saisana, M. & Tarantola, S. in *Global Sensitivity Analysis: The Primer* 109–154 (2008). at <http://doi.wiley.com/10.1111/j.1751-5823.2008.00062_17.x>
9. Saini, A., Clarke, J., Jariyasopit, N., Rauert, C., Schuster, J. K., Halappanavar, S., Evans, G. J., Su, Y. & Harner, T. Flame retardants in urban air: A case study in Toronto targeting distinct source sectors. *Environmental Pollution* **247,** 89–97 (2019).
10. Kurt-Karakus, P., Alegria, H., Birgul, A., Gungormus, E. & Jantunen, L. Organophosphate ester (OPEs) flame retardants and plasticizers in air and soil from a highly industrialized city in Turkey. *Science of The Total Environment* **625,** 555–565 (2018).
11. Salamova, A., Hermanson, M. H. & Hites, R. A. Organophosphate and halogenated flame retardants in atmospheric particles from a European Arctic site. *Environ. Sci. Technol.* **48,** 6133–6140 (2014).
12. OpenAI. DALL·E 2. (2022). at <https://openai.com/dall-e-2/>
